# Association of pulmonary artery catheter with in-hospital outcomes after cardiac surgery in the United States: National Inpatient Sample 1999–2019

**DOI:** 10.1038/s41598-023-40615-6

**Published:** 2023-08-19

**Authors:** Hind A. Beydoun, May A. Beydoun, Shaker M. Eid, Alan B. Zonderman

**Affiliations:** 1 Department of Research Programs, Fort Belvoir Community Hospital, 9300 DeWitt Loop, Fort Belvoir, VA 22060 USA; 2 Laboratory of Epidemiology and Population Sciences, National Institute on Aging Intramural Research Program, Baltimore, Maryland 21224 United States; 3 Department of Medicine, Johns Hopkins University School of Medicine, Baltimore, Maryland 21224 United States

**Keywords:** Health care, Medical research, Risk factors, Diseases, Cardiovascular diseases

## Abstract

To examine associations of pulmonary artery catheter (PAC) use with in-hospital death and hospital length of stay (days) overall and within subgroups of hospitalized cardiac surgery patients. Secondary analyses of 1999–2019 National Inpatient Sample data were performed using 969,034 records (68% male, mean age: 65 years) representing adult cardiac surgery patients in the United States. A subgroup of 323,929 records corresponded to patients with congestive heart failure, pulmonary hypertension, mitral/tricuspid valve disease and/or combined surgeries. We evaluated PAC in relation to clinical outcomes using regression and targeted maximum likelihood estimation (TMLE). Hospitalized cardiac surgery patients experienced more in-hospital deaths and longer stays if they had ≥ 1 subgroup characteristics. For risk-adjusted models, in-hospital deaths were similar among recipients and non-recipients of PAC (odds ratio [OR] 1.04, 95% confidence interval [CI] 0.96, 1.12), although PAC was associated with more in-hospital deaths among the subgroup with congestive heart failure (OR 1.14, 95% CI 1.03, 1.26). PAC recipients experienced shorter stays than non-recipients (β =  − 0.40, 95% CI − 0.64, − 0.15), with variations by subgroup. We obtained comparable results using TMLE. In this retrospective cohort study, PAC was associated with shorter stays and similar in-hospital death rates among cardiac surgery patients. Worse clinical outcomes associated with PAC were observed only among patients with congestive heart failure. Prospective cohort studies and randomized controlled trials are needed to confirm and extend these preliminary findings.

## Introduction

Pulmonary artery catheters (PAC) generate unique hemodynamic data that can guide therapeutic decision-making in cardiac surgery. Observational studies have suggested that their use may trigger higher intensity perioperative treatments without translating into improved outcomes^1–3^. In the absence of data from randomized trials, international society guidelines have made weak recommendations discouraging their routine use while acknowledging both a potential role and a lack of evidence in specific cases^4,5^. Ongoing uncertainty has resulted in widespread variation in international practices^6–8^, and the role of PAC remains controversial but highly editorialized^9,10^.

A PAC is used to generate direct (central venous pressure (CVP), right atrial pressure (RAP), right ventricle pressure (RVP), pulmonary artery pressure (PAP), pulmonary artery occlusion pressure (PAOP), cardiac output (CO), mixed venous oxyhemoglobin saturation (SvO_2_)) and indirect (systemic vascular resistance (SVR), pulmonary vascular resistance (PVR), cardiac index (CI), stroke volume index (SVI), left ventricular stroke work index (LVSWI), right ventricular stroke work index (RVSWI), oxygen delivery (DO_2_), oxygen uptake (VO_2_)) measurements, for the purpose of guiding treatment selection as well as monitoring response to treatment for pre-existing and ongoing chronic conditions. The accuracy and clinical relevance of these hemodynamic data have been previously questioned^10–12^. For instance, estimates of CO derived from PAC is subject to error related to technique, miscalculation, cardiac abnormalities, patient posture, and extra-cardiac abnormalities. A review of the literature suggested that CO must change by at least 25% to be detected by a PAC^9^.

There is currently limited evidence from single-center or multi-center retrospective and prospective cohort studies suggesting that clinical outcomes such as in-hospital mortality, intensive care unit (ICU) admission, length of hospital stay, duration of mechanical ventilation, inotrope use, acute kidney injury and infection, may differ between recipients and non-recipients of PAC in the context of cardiac surgery^1–3,6–8,13–15^. Whereas these observational studies have yielded inconsistent findings, few of these studies have applied causal modeling strategies or were sufficiently large to stratify cardiac surgery patients into distinct subgroups when comparing PAC recipients and non-recipients on clinical outcomes. Accordingly, a gap in knowledge exists whereby the utility of PAC in subgroups of patients having congestive heart failure, pulmonary hypertension, mitral/tricuspid valve disease or combined surgeries, remains under-studied^6^. Studies that rely on large administrative databases can overcome this issue by leveraging national samples. The objective of this retrospective cohort study was to examine the association of PAC receipt with clinical outcomes among hospitalized cardiac surgery patients, both overall and within subgroups, using the 1999–2019 National Inpatient Sample (NIS), risk-adjustment as well as causal [targeted maximum likelihood estimation (TMLE)] models.

## Methods

### Data source

Secondary analyses of existing data from the Agency for Healthcare Research and Quality (AHRQ) Healthcare Cost and Utilization Project (HCUP) NIS were performed for the time-period of 1999–2019. The NIS is the largest publicly available, all-payer inpatient care database of community (non-federal) hospitals in the U.S. It consists of 5–8 million hospital discharge records sampled from 1000 hospitals on an annual basis since 1988. Each year, a 20% stratified probability sample of hospitals (before 2012) or hospital discharge records (since 2012) were selected from all participating HCUP states, stratified by bed size, teaching status, urbanicity, and region. Within the NIS database, hospital discharge weights were provided that can be used to generate national estimates, for all years combined, and to examine trends over time taking into consideration sampling design changes in 2012. The NIS data elements included patient demographics, up to 15 diagnoses and 15 procedures, as well as hospital course and outcomes. All methods were carried out in accordance with relevant guidelines and regulations. The study was determined to be research not involving human subjects at Fort Belvoir Community Hospital. Since no experiments on humans and/or use of human tissue were performed as part of this study, a waiver of institutional review board approval was granted at Fort Belvoir Community Hospital. Due to the nature of the research study, informed consent was waived by the Institutional Review Board of Fort Belvoir Community Hospital. Supplemental Digital Content 1 provides a listing of all ICD-9-CM/ICD-10 diagnostic and procedure codes applied to define eligibility criteria and variables of interest. Of note, ICD-9-CM codes were used for the years 1999–2014 and the first three quarters of the year 2015. By contrast, ICD-10 codes were used for the fourth quarter of the year 2015 and the years 2016–2019.

### Eligibility criteria

**Figure 1:**
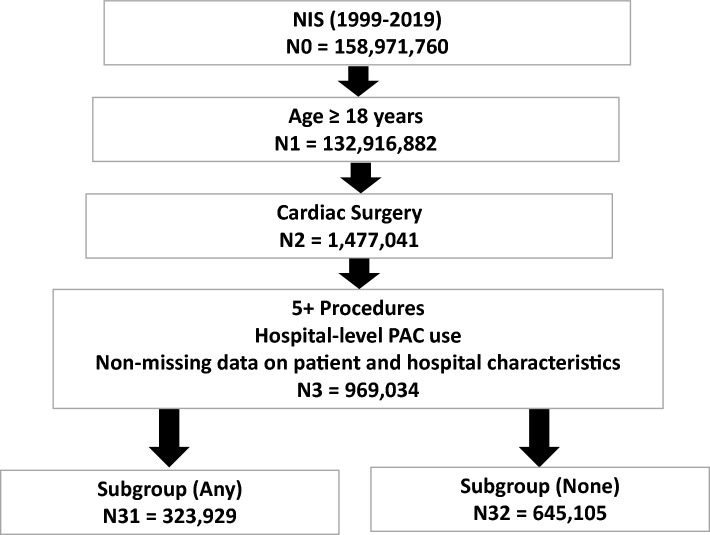
Study flowchart—National Inpatient Sample (1999–2019).

Variables pertaining to patient and hospital characteristics, diagnostic and procedure codes, and outcomes were defined using data elements from the NIS core and hospital databases, after applying pre-defined inclusion and exclusion criteria. The study population met the following inclusion criteria: (1) Age ≥ 18 years; (2) At least one of the first 15 procedure variables (PR1-PR15) comprise an ICD-9-CM/ICD-10 code that corresponds to cardiac surgery. A comprehensive list of ICD-9-CM/ICD-10 procedure codes that correspond to cardiac surgery was compiled and hospital discharge records were selected if the patient had at least one type of cardiac surgery. After excluding hospitals with < 50 cardiac surgeries performed overall between 1999 and 2019 and those with zero PACs per year, patients were excluded if they met at least one of the following criteria: (1) < 5 procedure codes; (2) missing data on patient and hospital characteristics described below. We chose to exclude hospital records with less than 5 procedure codes to maximize the total sample size while minimizing the likelihood of misclassifying patients who underwent multiple types of cardiac surgery. As show in Fig. 1, 132,916,882 of 158,971,760 1999–2019 NIS records corresponded to patients ≥ 18 years of age, and of those 1,477,041 records involved cardiac surgeries. After applying exclusion criteria, 969,034 records (68% male, mean age: 65 years) were kept (92,159 PAC recipients and 876,875 PAC non-recipients), of which 323,929 corresponded to patients that had one or more of the subgroup characteristics (32,539 PAC recipients and 291,390 PAC non-recipients) and 645,105 corresponded to patients who did not have any of the subgroup characteristics (59,620 PAC recipients and 585,485 PAC non-recipients).

### Patient characteristics

Patient-level characteristics were defined as age (in ‘years’), sex (“Male”, “Female”), race/ethnicity (“White”, “African American”, “Hispanic”, “Other”), Charlson comorbidity index [CCI] (“0”, “1”, “2+”), elective admissions (“Yes”, “No”), admission quarter (“1st quarter”, “2nd quarter”, “3rd quarter”, “4th quarter”), weekend admission status (“Monday–Friday”, “Saturday–Sunday”) and primary payer (“Medicare”, “Medicaid”, “Private insurance”, “Self-pay”, “No pay”, “Other”). The CCI score reflects the cumulative increase in likelihood of one-year mortality due to the severity of the effect of comorbidities. In this study, the CCI was calculated using 15 ICD-9-CM/ICD-10 diagnostic codes by the Stata command *charlson*. Alternatively, the CCI was replaced with dichotomous (“Yes”, “No”) variables based on ICD-9-CM/ICD-10 diagnostic codes, representing each of the following comorbidities that are relevant to cardiovascular surgery patients: congestive heart failure, myocardial infarction, aortic valve disease, mitral valve disease, tricuspid valve disease, pulmonary valve disease, respiratory failure, chronic obstructive pulmonary disease, pulmonary hypertension, hypertension, pneumonia, atherosclerotic disease, stroke, diabetes, cancer, and chronic liver disease.

### Hospital characteristics

Hospital-level characteristics were defined as hospital region (“Northeast”, “Midwest”, “South”, “West”), hospital control (“Government or Private”, “Government, non-federal”, “Private, not-for-profit”, “Private, investor-owned”, “Private”), hospital location and teaching status (“Rural”, “Urban—Non-Teaching”, “Urban—Teaching”) and hospital bed size (“Small”, “Medium”, “Large”).

### Selected subgroups

ICD-9-CM/ICD-10 diagnostic and procedure codes were used to define specific subgroups: “heart failure”, “pulmonary hypertension”, “mitral or tricuspid valve disease” and “combined surgery”. The “combined surgery” subgroup consists of hospital records whereby at least two of the following procedures were applied: Coronary Artery Bypass Grafting (CABG), aortic valve surgery, mitral valve surgery, tricuspid valve surgery, pulmonary valve surgery. Furthermore, hospital discharge records were defined as “Any” versus “None” based on presence or absence of at least one of these characteristics.

### PAC receipt

PAC receipt was defined as the study exposure of interest. A comprehensive list of ICD-9-CM/ICD-10 procedure codes that correspond to PAC receipt were generated for eligible hospitalizations in the 1999–2019 NIS database. These codes were obtained from primary and secondary procedure variables and, as such, hospital discharge records were classified into two categories: (1) PAC recipient; (2) PAC non-recipient. Furthermore, hospital-level PAC rates were calculated and categorized into quartiles for the purpose of sensitivity analyses.

### Clinical outcomes

Data were extracted on multiple study outcomes, including in-hospital mortality (“deceased” vs. “alive”) and in-hospital LOS (days). Total LOS was defined as a continuous variable in the context of regression modeling and categorized as “≥ 7 days” vs. “< 7 days” in the context of regression and causal modeling.

### Statistical analysis

All statistical analyses were conducted using Stata version 17 (StataCorp, College Station, TX), taking into consideration complex sampling design as well as specific recommendations^16^. Descriptive statistics included mean (± standard error) for continuous variables and frequencies with percentages for categorical variables. Bivariate associations were examined using uncorrected Chi-square and design-based F-tests, as appropriate. Linear and binary logistic regression models were constructed to estimate crude and adjusted beta coefficients as well as odds ratios (cOR and aOR) with their 95% confidence intervals (CI) for exposure variables as predictors of the selected health outcomes. Risk-adjustment and targeted maximum likelihood estimation (TMLE) with Super Learner algorithms were performed when comparing recipients and non-recipients of PAC on health outcomes, overall, as well as according to pre-specified subgroups. Multivariable models were adjusted for age, sex, race/ethnicity, CCI, elective admissions, admission quarter, weekend admission status, primary payer, hospital region, hospital control, hospital location and teaching status as well as hospital bed size. Sensitivity analyses were performed whereby CCI was replaced with the comorbidities described above as covariates in overall risk-adjusted models. Because regression methods are frequently biased if outcome models are mis-specified, causal inference methods incorporating propensity scores, the G-formula or TMLE are preferred^17^. Although propensity score methods necessitate exposure models to be correctly specified, double-robust methods such as TMLE require correct specification of either outcome or exposure models^17^. TMLE is a semiparametric estimator allowing use of machine learning algorithms to minimize model misspecification^17^. Unlike TMLE, classical regression methods for estimating the average treatment effect (ATE), or risk difference, assume that ATE is constant across confounder levels with no effect modification. We applied the *eltmle* package in Stata, while using Super Learner with tenfold cross-validation to evaluate the predictive performance for potential outcomes and weighted averages as a propensity score for distinct machine learning algorithms. The default Super Learner machine learning algorithm was applied as previously defined in an R v.1.2.0-5 package: (1) stepwise selection, (2) generalized linear modeling (GLM), (3) GLM variant that includes second order polynomials and two-by-two interactions of main terms included in the model. The ATE, causal risk ratio (CRR) and marginal odds ratio (MOR) were estimated with 95% CI for each hypothesized relationship using TMLE^17–21^. Using risk-adjustment and TMLE analyses, we performed sensitivity analyses whereby the overall relationship between PAC use and the outcomes of interest were examined within quartiles of hospital-level PAC rates. Complete subject analyses were performed after examination of patterns of missingness. Two-sided statistical tests were conducted and P < 0.05 were considered statistically significant.

### Ethical approval

Since the project was determined to be research not involving human subjects, a waiver of institutional review board approval was granted at Fort Belvoir Community Hospital. Due to the nature of the research study, informed consent was waived by Institutional Review Board of Fort Belvoir Community Hospital. The project adhered to relevant ethical guidelines/regulations in accordance with the Declaration of Helsinki.

## Results

Table 1 presents patient and hospital characteristics according to subgroup and PAC statuses. In general, there were fewer disparities according to PAC receipt among patients having versus patients not having the selected subgroup characteristics. The overall rate of PAC receipt among hospitalized cardiac surgery patients was 9.49%, with a significantly higher PAC rate among patients with any versus none of subgroup characteristics (10.01% vs. 9.23%, P = 0.007).

**Table 1:** Patient and hospital characteristics according to subgroup and pulmonary artery catheter status—1999–2019 National Inpatient Sample (n = 969,034).

	Mean ± SEM or %					
	Subgroup status					
	Any* (n = 323,929)		None (n = 645,105)		Total (n = 969,034)	
	PAC recipient	PAC non-recipient	PAC recipient	PAC non-recipient	PAC recipient	PAC non-recipient
Age (years)	P = 0.92		P < 0.0001		P = 0.0001	
N	32,539	291,390	59,620	585,485	92,159	876,875
Mean ± SEM	67.2 ± 0.2	67.2 ± 0.1	65.0 ± 0.13	64.5 ± 0.08	65.80 ± 0.13	65.4 ± 0.08
Sex	P = 0.024		P < 0.0001		P = 0.064	
Male	61.2	61.9	71.2	71.1	67.6	68.0
Female	38.8	38.0	28.8	28.9	32.4	31.9
Race/ethnicity	P = 0.59		P = 0.027		P = 0.095	
White	66.6	66.4	65.8	64.7	66.1	65.3
African-American	6.1	6.6	4.2	5.1	4.8	5.6
Hispanic	5.6	5.8	5.3	5.8	5.4	5.8
Other	5.2	5.1	4.6	5.1	4.8	5.1
Unknown	16.5	16.1	20.0	19.3	18.8	18.3
Charlson comorbidity index	P = 0.53		P = 0.56		P = 0.52	
0	17.8	18.3	21.6	21.4	20.2	20.3
1	29.4	29.4	33.2	33.7	31.9	32.3
2 +	52.8	52.4	45.2	44.9	47.9	47.4
Comorbidities*						
Congestive heart failure	P = 0.47		–		P = 0.33	
Yes	31.4	31.9	0.0	0.0	11.0	10.7
No	68.6	68.0	100.0	100.0	88.9	89.3
Myocardial infarction	P = 0.0001		P < 0.0001		P < 0.0001	
Yes	6.9	5.9	10.9	9.3	9.5	8.2
No	93.0	94.1	89.0	90.7	90.5	91.8
Aortic valve disease	P = 0.20		P < 0.0001		P < 0.0001	
Yes	35.1	34.2	11.9	8.8	20.1	17.3
No	64.8	65.8	88.1	91.1	79.9	82.7
Mitral valve disease	P < 0.0001		–		P < 0.0001	
Yes	38.2	34.4	0.0	0.0	13.5	11.5
No	61.8	65.5	100.0	100.0	86.5	88.5
Tricuspid valve disease	P = 0.78		–		P = 0.19	
Yes	14.5	14.3	0.0	0.0	5.1	4.8
No	85.5	85.6	100.0	100.0	94.5	95.2
Pulmonary vascular disease	P = 0.05		P = 0.001		P = 0.003	
Yes	0.3	0.4	0.06	0.1	0.2	0.05
No	99.7	99.6	99.9	99.9	99.8	99.9
Respiratory failure	P = 0.01		P = 0.37		P = 0.09	
Yes	15.9	14.7	11.0	10.6	12.8	11.9
No	84.1	85.3	88.9	89.4	87.2	88.0
Chronic obstructive pulmonary disease	P < 0.0001		P < 0.0001		P < 0.0001	
Yes	26.2	21.1	12.8	10.7	17.6	14.2
No	73.8	78.8	87.1	89.2	82.4	85.8
Pulmonary hypertension	P < 0.0001		–		P < 0.0001	
Yes	18.2	14.2	0.0	0.0	6.4	4.7
No	81.8	85.8	100.0	100.0	93.6	95.3
Hypertension	P < 0.0001		P = 0.001		P = 0.0001	
Yes	3.1	4.6	0.0	7.6	4.7	6.6
No	96.8	95.3	100.0	92.4	95.3	93.4
Pneumonia	P = 0.01		P = 0.21		P = 0.03	
Yes	5.4	4.9	3.6	3.5	4.3	4.0
No	94.6	95.0	96.3	96.5	95.7	96.0
Atherosclerotic disease	P = 0.99		P = 0.91		P = 0.96	
Yes	0.02	0.02	0.01	0.01	0.02	0.02
No	99.9	99.9	99.9	99.9	99.9	99.9
Stroke	P = 0.21		P = 0.85		P = 0.42	
Yes	0.2	0.3	0.2	0.2	0.2	0.3
No	99.8	99.7	99.8	99.8	98.2	99.7
Diabetes	P < 0.0001		P = 0.0006		P = 0.0001	
Yes	1.5	2.2	1.4	1.9	1.4	2.0
No	98.5	97.8	98.6	98.0	98.5	97.9
Cancer	P = 0.73		P = 0.40		P = 0.72	
Yes	2.1	2.0	1.8	1.9	1.9	1.9
No	97.9	97.9	98.2	98.1	98.1	98.1
Chronic liver disease	P = 0.0006		P = 0.28		P = 0.01	
Yes	2.4	1.9	1.4	1.3	1.7	1.5
No	9.8	98.0	98.6	98.7	98.2	98.5
Elective admission	P = 0.082		P < 0.0001		P < 0.0001	
Yes	51.3	49.9	48.0	40.9	49.2	43.6
No	48.7	50.0	51.9	59.5	50.8	56.4
Admission quarter	P = 0.098		P = 0.035		P = 0.018	
1st quarter	25.7	25.2	26.1	25.3	25.9	25.2
2nd quarter	25.9	25.9	25.7	25.4	25.8	25.6
3rd quarter	24.1	23.9	24.0	24.3	24.1	24.2
4th quarter	24.2	24.9	24.1	24.9	24.2	24.9
Weekend admission	P = 0.19		P < 0.0001		P < 0.0001	
Monday–Friday	90.1	89.7	89.5	87.8	89.7	88.5
Saturday–Sunday	9.9	10.3	10.5	12.2	10.3	11.5
Primary payer	P = 0.26		= 0.0001		= 0.0036	
Medicare	61.9	62.6	52.8	51.8	56.0	55.4
Medicaid	6.7	6.4	5.9	6.1	6.2	6.2
Private	26.6	26.0	35.5	35.6	32.4	32.4
Self-pay	2.3	2.5	2.7	3.4	2.6	3.1
No pay	0.3	0.3	0.5	0.4	0.4	0.3
Other	0.2	2.1	2.6	2.7	0.2	2.5
Region	P = 0.026		P = 0.093		P = 0.072	
Northeast	19.9	21.8	19.5	18.3	19.7	19.5
Midwest	28.8	23.2	29.6	23.5	29.3	23.4
South	32.7	37.6	35.4	42.2	34.5	40.7
West	18.6	17.4	15.4	15.9	16.6	16.5
Location and teaching status	P = 0.080		P = 0.38		P = 0.27	
Rural	3.5	23.4	4.7	3.6	4.3	3.2
Urban—non-teaching	28.3	27.5	32.9	31.9	31.2	30.5
Urban—teaching	68.4	70.2	62.4	64.4	64.5	66.4
Bed size	P = 0.99		P = 0.69		P = 0.85	
Small	6.4	6.4	5.5	5.9	5.8	6.3
Medium	18.5	18.7	19.8	19.6	19.3	19.3
Large	75.1	74.9	74.7	74.7	74.8	74.4

*PAC* pulmonary artery catheter, *SEM* standard error of the mean.

*Includes congestive heart failure, pulmonary hypertension, mitral/tricuspid valve repair and combined surgeries.

An in-hospital mortality rate of 4.05% was estimated in the overall cardiac surgery patient population, with significant differences based on subgroup characteristics (none: 3.05% vs. any: 6.06%, P < 0.0001). Similarly, the average length of stay was estimated at 11.40 days, with significantly higher levels (P < 0.0001) among cardiac surgery patients with any (13.37 days) versus none (10.41 days) of the subgroup characteristics. Similar disparities were observed according to presence of specific characteristics, namely, congestive heart failure, pulmonary hypertension, mitral or tricuspid valve disease and combined surgeries (Table 2).

**Table 2:** Summary statistics for in-hospital death and hospital length of stay according to subgroup status—1999–2019 National Inpatient Sample (n = 969,034).

	In-hospital Death	Hospital Length of stay
	%	Mean (SEM)
Overall	4.05%	11.40 (± 0.06)
Subgroup status	P < 0.0001	P < 0.0001
None	3.05%	10.41 (± 0.05)
Any*	6.06%	13.37 (± 0.08)
Congestive heart failure	P < 0.0001	P < 0.0001
Yes	3.83%	14.42 (± 0.08)
No	5.94%	11.04 (± 0.06)
Pulmonary hypertension	P < 0.0001	P < 0.0001
Yes	4.00%	12.99 (± 0.11)
No	5.25%	11.32 (± 0.06)
Mitral/tricuspid valve repair	P < 0.0001	P < 0.0001
Yes	3.75%	13.18 (± 0.12)
No	5.85%	11.10 (± 0.05)
Combined surgeries	P < 0.0001	P < 0.0001
Yes	3.38%	13.18 (± 0.11)
No	7.62%	10.92 (± 0.05)

*SEM* standard error of the mean.

*Defined based on ICD-9 or ICD-10 diagnostic or procedure codes for congestive heart failure, pulmonary hypertension, mitral/tricuspid valve repair, and/or combined surgeries as described in the Supplemental Materials.

**Figure 2:**
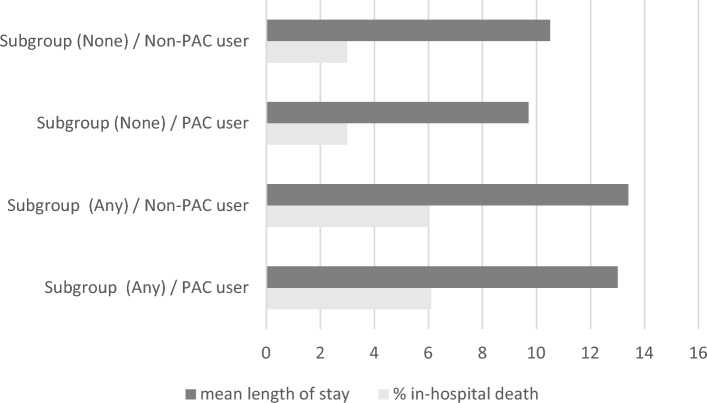
In-hospital death and length of hospital stay among recipients and non-recipients of pulmonary artery catheter, overall, and according to subgroup status—National Inpatient Sample (1999–2019).

**Figure 3:**
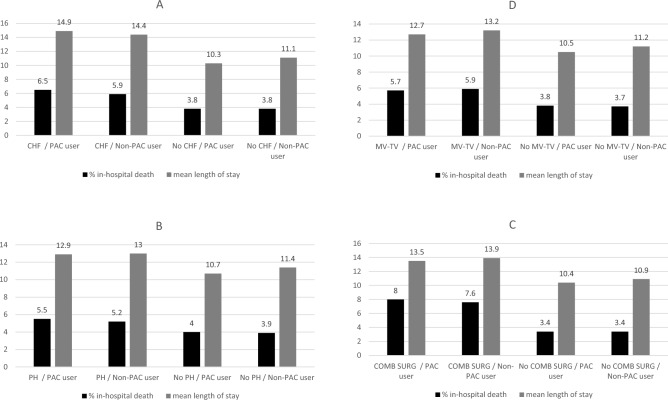
In-hospital death and length of hospital stay among recipients and non-recipients of pulmonary artery catheter among specific groups—National Inpatient Sample (1999–2019). (**A**) Congestive heart failure; (**B**) pulmonary hypertension; (**C**) mitral or tricuspid valve disease; (**D**) combined surgeries.

Crude analyses suggested significant differences in hospital LOS (10.85 (± 0.12) days vs. 11.46 (± 0.06) days, P < 0.0001), but not in-hospital mortality rates (4.12% vs. 4.05%, P = 0.63), when comparing PAC recipients to non-recipients. The unadjusted and risk-adjusted regression models for PAC receipt as a predictor of in-hospital mortality and hospital length of stay by subgroup are presented in Table 3 and summary statistics are displayed Figs. 2 and 3. In general, the odds of in-hospital death were not significantly different between PAC recipients and non-recipients, after controlling for patient and hospital characteristics (OR 1.04, 95% CI 0.96, 1.12). In risk-adjusted models, PAC recipients experienced shorter hospitalizations (β =  − 0.40, 95% CI − 0.64, − 0.15) than non-recipients; after stratifying by subgroup, PAC was associated with longer hospital stays (β = 0.68, 95% CI 0.31, 1.07) among patients with congestive heart failure, but was not associated with hospital LOS among patients with pulmonary hypertension (β =  − 0.032, 95% CI − 0.44, 0.38) in risk-adjusted models. Similarly, the odds of experiencing hospital LOS ≥ 7 days was significantly less among PAC recipients versus non-recipients (OR 0.77, 95% CI 0.72, 0.82). Similar results were obtained when CCI was replaced with comorbidities in the overall risk-adjusted model (Table S.1).

**Table 3:** Unadjusted and risk-adjusted linear and logistic regression models for pulmonary artery catheter receipt as a predictor of in-hospital death and hospital length of stay by subgroup status—1999–2019 Nationwide Inpatient Sample (n = 969,034).

	PAC use (yes vs. no)		
	In-hospital death Deceased vs. alive	Hospital Length of stay (days)	Hospital Length of stay ≥ 7 days vs. < 7 days
	OR (95% CI)	β (95% CI)	OR (95% CI)
Unadjusted models			
Overall	1.02 (0.94, 1.10)	− 0.60 (− 0.86, − 0.35)	0.75 (0.71, 0.80)
Subgroup status			
None	0.99 (0.91, 1.09)	− 0.81 (− 1.01, − 0.60)	0.71 (0.67, 0.76)
Any	1.01 (0.94, 1.09)	− 0.39 (− 0.72, − 0.08)	0.81 (0.76, 0.87)
Congestive heart failure	1.12 (1.01, 1.23)	0.63 (0.23, 1.01)	0.84 (0.77, 0.91)
Pulmonary hypertension	1.06 (0.91, 1.22)	− 0.087 (− 0.51, 0.34)	0.83 (0.75, 0.92)
Mitral/tricuspid valve repair	0.97 (0.88, 1.08)	− 0.54 (− 0.97, − 0.10)	0.78 (0.71, 0.85)
Combined surgeries	1.06 (0.97, 1.16)	− 0.46 (− 0.83, − 0.10)	0.89 (0.83, 0.96)
Risk-adjusted models*			
Overall	1.04 (0.96, 1.12)	− 0.40 (− 0.64, − 0.15)	0.77 (0.72, 0.82)
Perioperative risk			
None	1.03 (0.94, 1.13)	− 0.58 (− 0.78, − 0.38)	0.74 (0.69, 0.79)
Any	1.01 (0.95, 1.09)	− 0.26 (− 0.56, 0.037)	0.81 (0.76, 0.87)
Congestive heart failure	1.14 (1.03, 1.26)	0.68 (0.31, 1.07)	0.85 (0.78, 0.92)
Pulmonary hypertension	1.05 (0.91, 1.22)	− 0.032 (− 0.44, 0.38)	0.83 (0.74, 0.91)
Mitral/tricuspid valve repair	0.98 (0.88, 1.08)	− 0.38 (− 0.75, − 0.021)	0.78 (0.71, 0.85)
Combined surgeries	1.06 (0.96, 1.16)	− 0.40 (− 0.76, − 0.04)	0.88 (0.83, 0.95)

*CI* confidence interval, *OR* odds ratio, *PAC* pulmonary artery catheter.

*Adjusted for age, sex, race/ethnicity, Charlson’s comorbidity index, elective admissions, admission quarter, weekend admission status, primary payer, hospital region, hospital control, hospital location and teaching status and hospital bed size.

Table 4 presents ATE, MOR and CRR with their 95% CI for TMLE-based causal models for relationships of PAC receipt with in-hospital death and hospital LOS, overall and within subgroups. In general, the results of these causal models were comparable to those of risk-adjusted models, although PAC receipt was significantly associated with in-hospital death (ATE = 0.0018, MOR = 1.05, CRR = 1.05), and this result is likely driven by congestive heart failure patients (ATE = 0.0071, MOR = 1.13, CRR = 1.12). Similarly, PAC recipients were less likely to experience hospital LOS ≥ 7 days (ATE = − 0.0422, MOR = 0.81, CRR = 0.94). As suggested by non-overlapping 95% CI for MOR and COR estimates, a significantly stronger inverse relationship between PAC receipt and prolonged hospitalization was observed among patients with none of the selected subgroup characteristics as compared to those with at least one of these characteristics. Stratified analyses based on subgroup status did not reveal any other heterogeneity in the hypothesized relationships between PAC and clinical outcomes.

**Table 4:** Causal models using targeted maximum likelihood estimation for pulmonary artery catheter receipt as a predictor of in-hospital death and hospital length of stay by subgroup status—1999–2019 National Inpatient Sample (n = 969,034).

	PAC use	
	In-hospital death Deceased vs. alive	Hospital Length of stay ≥ 7 days vs. < 7 days
Average treatment effect (95% CI)		
Overall	0.0018 (0.0003, 0.0032)	− 0.0422 (− 0.0451, − 0.0392)
Subgroup status		
None	0.0006 (− 0.0010, 0.0022)	− 0.0505 (− 0.0543, − 0.046)
Any	− 0.0009 (− 0.0019, 0.0037)	− 0.0311 (− 0.0356, − 0.0266)
Congestive heart failure	0.0071 (0.0020, 0.0122)	− 0.0215 (− 0.0287, − 0.0142)
Pulmonary hypertension	0.000 (− 0.0061, 0.0062)	− 0.0255 (− 0.0355, − 0.0154)
Mitral/tricuspid valve repair	− 0.0009 (− 0.0049, 0.0031)	− 0.0397 (− 0.0464, − 0.0330)
Combined surgeries	0.0033 (− 0.0015, 0.0081)	− 0.0186 (− 0.0254, − 0.0117)
Marginal odds ratio (95% CI)		
Overall	1.05 (1.01, 1.08)	0.81 (0.80, 0.83)
Subgroup status		
None	1.02 (0.97, 1.08)	0.79 (0.78, 0.81)
Any	1.02 (0.97, 1.07)	0.83 (0.81, 0.85)
Congestive heart failure	1.13 (1.03, 1.22)	0.86 (0.81, 0.90)
Pulmonary hypertension	1.01 (0.89, 1.14)	0.84 (0.79, 0.90)
Mitral/tricuspid valve repair	0.98 (0.91, 1.06)	0.80 (0.77, 0.83)
Combined surgeries	1.05 (0.98, 1.12)	0.90 (0.86, 0.93)
Causal risk ratio (95% CI)		
Overall	1.05 (1.01, 1.08)	0.94 (0.94, 0.95)
Subgroup status		
None	1.02 (0.97, 1.08)	0.93 (0.92, 0.93)
Any	1.02 (0.97, 1.06)	0.96 (0.96, 0.97)
Congestive heart failure	1.12 (1.04, 1.21)	0.97 (0.97, 0.98)
Pulmonary hypertension	1.01 (0.90, 1.14)	0.97 (0.96, 0.98)
Mitral/tricuspid valve repair	0.99 (0.92, 1.06)	0.95 (0.94, 0.96)
Combined surgeries	1.04 (0.98, 1.11)	0.98 (0.97, 0.99)

Adjusted for age, sex, race/ethnicity, Charlson’s comorbidity index, elective admissions, admission quarter, weekend admission status, primary payer, hospital region, hospital control, hospital location and teaching status and hospital bed size.

*CI* confidence interval, *PAC* pulmonary artery catheter.

Sensitivity analyses were performed whereby unadjusted, risk-adjusted, and TMLE-adjusted models were constructed after stratifying the study population according to quartiles of hospital-level PAC rates (Fig. S.1, Tables S.2, S.3). These analyses revealed trends that are distinct from those presented in Tables 3 and 4. In particular, hospitalizations whereby PAC rates were in the first, second or third quartiles experienced more in-hospital deaths with either no difference or longer stays among PAC recipients versus PAC non-recipients. By contrast, hospitalizations whereby PAC rates were in the fourth quartile had fewer in-hospital deaths with shorter stays among PAC recipients versus PAC non-recipients. Furthermore, the magnitude of associations between PAC use and outcomes of interest declined between the first and third quartile of hospital-level PAC rates. After excluding combined surgeries, we separately examined subgroups of cardiac surgery patients who underwent mitral valve or tricuspid valve repairs. As shown in Table S.4, PAC use was not significantly related to in-hospital death in either subgroup but was related to shorter hospitalizations among the mitral valve repair group and longer hospitalizations among the tricuspid valve repair group.

## Discussion

In this retrospective cohort study using a representative sample of 969,034 1999–2019 NIS records corresponding to hospitalized cardiac surgery patients from the United States, we applied risk adjustment and causal modeling to examine PAC receipt in relation to clinical outcomes, overall, and according to selected subgroup characteristics. Our results suggested similar in-hospital death rates and shorter hospital stays among PAC recipients versus non-recipients. Heterogeneities by PAC receipt were found mostly among patients with congestive heart failure, whereby PAC recipients experienced worse clinical outcomes compared to non-recipients. These findings were consistent with some, but not all, similarly conducted studies. For instance, Chiang et al*.* utilized the NIS database and included 2,063,227 cardiac surgery cases from 2000 to 2010^7^. In this study, PAC receipt was associated with increased in-hospital mortality, risk of prolonged mechanical ventilation and risk of hospitalizations greater than 30 days. Conversely, a study by Brovman et al*.* utilized the US National Anesthesia Clinical Outcomes Registry to analyze data from 116,333 cardiac surgery cases and found that PAC receipt was associated with a reduction of intraoperative red blood cell transfusion but not with operative mortality^13^. Inconsistent findings among observational studies in the literature with respect to clinical outcomes after PAC receipt are likely due to methodological differences among these studies, highlighting the need for randomized controlled trials to elucidate the risks and benefits associated with PAC use.

Although considerably lower than PAC rates estimated among older studies focused on cardiac surgery patients ranging between 25 and 69%^1,2,6,8,13,15,22^, the estimated PAC rate of approximately 9% among cardiac surgery patients in this study was nearly 10 times that reported by Ikuta et al. using 1999–2013 Medicare data^23^ and Weiner et al. using 1993–2004 NIS data^24^, a more general population of hospitalized patients. It was also considerably higher than NIS estimates of PAC use among congestive heart failure^25–27^ and aneurysmal subarachnoid hemorrhage^28^ hospitalizations, regardless of whether or not cardiac surgery was performed during hospitalization. However, this estimate was consistent with a recently published study by Vallabhajosyula et al*.*, whereby 2000–2014 NIS data on acute myocardial infarction-cardiogenic shock patients were analyzed, resulting in an estimated PAC rate of 8.1%^29^.

The utilization of PAC for the generation of hemodynamic data through direct and indirect measurements remains controversial. To date, this strategy aimed at enhancing recovery and reducing ICU LOS has not been adequately evaluated for cardiac surgery. A multicenter randomized controlled trial (RCT) including 1994 high-risk non-cardiac surgery patients found that PAC use had no effect on postoperative mortality but increased the risk of pulmonary embolism^30^. A small trial focused on high-risk combined valve surgeries in 40 patients compared hemodynamic goal-directed therapy with PAC to a transpulmonary thermodilution technique involving a central venous catheter. Investigators found that PAC was associated with an increased duration of mechanical ventilation, but similar length of stay in the ICU and hospital^31^. These findings from randomized controlled trials involving non-cardiac surgery patients are consistent with this study’s finding that PAC use may not be related to mortality risk, but are inconsistent with this study’s finding that PAC use may reduce length of stay, although heterogeneities according to underlying condition, type of surgery, and institution-wide prevalence of PAC use were also revealed by our analyses of the NIS database.

In this study, although in-hospital death and hospital LOS differed significantly among subgroups, the relationship between PAC and these clinical outcomes, for the most part, did not vary according to presence or absence of characteristics defining these subgroups. The finding that cardiac surgery patients with congestive heart failure who received a PAC might experience prolonged hospitalizations with more deaths requires further investigation. However, it is plausible that PAC is frequently used among patients with severe decompensated heart failure, and this could explain the observation that rates of in-hospital death were higher in the heart failure subgroup who had a PAC since PAC use is a surrogate for disease acuity and severity in this subgroup.

Similarly, the finding that hospital-level PAC rate might influence the relationship between PAC use and in-hospital outcomes may be interpreted in two different ways. First, PAC may be more beneficial in the context of hospitals with experience in PAC use. Second, hospital reporting of ICD codes for PAC use may be a marker for better quality healthcare, resulting in better outcomes among PAC recipients versus non-recipients. On the other hand, findings from risk-adjusted models were mostly comparable to those of TMLE-based causal models, suggesting that PAC use is likely not causally associated with poor clinical outcomes, but could potentially affect resource utilization within the hospital. These findings were comparable to some but not all previously conducted studies on cardiac surgery patients, of which only a few applied a methodology similar to TMLE, namely, propensity score matching (PSM). A recent PSM analysis published by Brown et al*.* included 11,820 patients undergoing coronary or valvular surgery and found that PAC was independently associated with prolonged ICU stay and packed red blood cell transfusion, but not with operative mortality^6^. Schwann et al*.* conducted a prospective cohort study of 5065 patients undergoing CABG with PSM. PAC receipt was independently associated with in-hospital mortality, ICU stay > 4 days, inotrope use, longer duration of mechanical ventilation and acute kidney injury^1^. Ramsey et al. studied 13,906 non-emergent CABG cases across 56 hospitals and found PAC receipt was independently associated with in-hospital mortality and increased ICU length of stay^8^. Resano et al. reported a single-center study involving 2414 off-pump CABG cases^2^. In this study, PAC receipt was associated with inotrope use, but not with in-hospital mortality or ICU length of stay > 4 days^2^. Similarly, a single center retrospective study by Xu et al*.* found PAC receipt was associated with inotrope requirement, but not with in-hospital mortality or acute kidney injury^3^.

Study results should be interpreted with caution in light of several limitations. First, secondary analyses that rely on administrative data are frequently limited in granularity and their ability to define exposures, outcomes and potential confounders and/or effect modifiers^14^. A PAC per se is not therapeutic but only a surrogate for therapeutic guidance and measurement of response to therapy. A patient may receive a PAC and still not receive the optimal treatment or be effectively managed. Ideally, the outcome of PAC use should include a change in hemodynamic measurements between baseline and follow-up time points in response to various therapies. However, these data are not available in this large administrative database. Unlike disease registries, the NIS database does not consistently collect detailed data on baseline characteristics of cardiac surgery patients. For instance, prognostic factors such as ejection fraction, disease acuity at presentation as well as duration and complexity of surgery or duration of PAC use were not consistently collected throughout the 20-year study period. Also, the NIS database consists of hospitalization records rather than unique patients, whereby hospital re-admissions cannot be ascertained. Second, complete subject analysis was performed with the potential for selection bias because of missing data. Selection bias may also result from more invasive monitoring by more severe cases with more complex surgeries. Third, many study variables were defined using ICD-9-CM/ICD-10 diagnostic and procedure codes, potentially leading to misclassification. Further research is needed to quantify the sensitivity and specificity of procedure codes used to define the use of medical devices such as PAC within administrative databases such as the NIS. Fourth, residual confounding could not be ruled out as an alternative explanation given the observational study design and the limited availability of data elements within the NIS database. Similarly, the role of chance could not be ruled out given the limited number of patients within specific subgroups for cardiac surgery. Larger databases are needed to distinguish hospitalization outcomes associated with PAC use among specific types of cardiac surgeries with different propensity to use a PAC, including CABG versus left ventricular assist device, or different types of mitral valve and tricuspid valve diseases. Fifth, the retrospective cohort design may not allow clear establishment of temporality between variables of interest, especially with regards to occurrence of cardiac surgery, PAC insertion and length of stay. As such, we were not able to evaluate complications such as acute kidney injuries, embolisms or infections that are potentially linked to PAC. Finally, time bias is plausible given that a culture shift may have occurred during this time period (1999–2019), with the majority of perioperative clinicians being more conservative in their use of PAC as compared to previous years (Fig. S.2). By the same token, the method by which PAC is inserted has changed over time, with more complications expected before ultrasound guided access became the standard of care. As such, study results could only be generalized to hospitalized patients within the period of interest. Further analyses suggested a significant decline in mortality (4.47% vs. 3.43%, P < 0.0001), with no significant differences in PAC use (9.68% vs. 9.21%, P = 0.56) or duration of hospitalization (11.38 days vs. 11.42 days, P = 0.71) among cardiac surgery patients between two decades (1999–2009 vs. 2010–2019). From a clinical perspective, substantially reduced in-hospital death rates after cardiac surgery with stable PAC rates over time suggests that improved cardiac surgery outcomes may be due to better pre-operative management and surgical techniques. Furthermore, PAC use may not have an impact on cardiac surgery outcomes unless the patient appropriately responds to titrations based on the PAC, and this cannot be ascertained with an administrative database such as the NIS. Although cardiac surgery patients have become more complex in recent years and PACs may enable clinicians to achieve better granularity in changing their hemodynamics, it is unclear if these changes would translate into better outcomes for these complex patients.

## Conclusions

In this retrospective cohort study involving a nationally representative sample of hospitalized cardiac surgery patients, PAC recipients and non-recipients experienced similar in-hospital death rates, with shorter hospital stays among PAC recipients versus non-recipients. These findings were mostly homogeneous across strata defined by selected characteristics, namely, pulmonary hypertension, mitral/tricuspid valve disease, and combined surgeries. PAC recipients experienced worse clinical outcomes compared to PAC non-recipients only among the subgroup of patients with congestive heart failure, with distinct findings among risk adjusted and TMLE analyses. These findings are consistent with some but not all previously conducted studies that tested similar hypotheses. The specific role of PAC in clinical outcomes after cardiac surgery remains uncertain. Therefore, prospective cohort studies and randomized controlled trials are needed to confirm and extend these preliminary findings.

### Supplementary Information

Supplementary Information.

## Data Availability

Since data used in this study were de-identified by the Agency for Healthcare Research and Quality, this study was considered research not involving human subjects by Fort Belvoir Community Hospital. The data that support the findings of this study are available from the Agency for Healthcare Research and Quality but restrictions apply to the availability of these data. These data are not considered public use since a data use agreement, completion of a specific training and purchase of data are necessary before data can be accessed. Data are however available from the lead author (HAB) upon reasonable request and with permission of the Agency for Healthcare Research and Quality.
