# Supplementary material for: Association of pulmonary artery catheter with in-hospital outcomes after cardiac surgery in the United States: National Inpatient Sample 1999–2019
Source: Sci Rep. 2023 Aug 19;13:13541. doi: 10.1038/s41598-023-40615-6 (PMC10439892; doi:10.1038/s41598-023-40615-6)
Supplement: Supplementary file 1 — Supplementary Information. [file 41598_2023_40615_MOESM1_ESM.docx]

**Supplemental Digital Content 1 – ICD-9/ICD-10 codes**

**CARDIOVASCULAR SURGERY:**

| **Type of surgery** | **ICD-9** | **ICD-10** |
| --- | --- | --- |
| **Coronary Artery Bypass Grafting** | **‘361’**  ***Means anything starting with ‘361’*** | **'210083', '210088', '210089', '021008C', '021008F', ‘021008W', '210093', '210098', '210099', '021009C', '021009F', '021009W', '02100A3', '02100A8', '02100A9', '02100AC', '02100AF', '02100AW', '02100J3', '02100J8', '02100J9', '02100JC', '02100JF', '02100JW', '02100K3', '02100K8', '02100K9', '02100KC', '02100KF', '02100KW', '02100Z3', '02100Z8', '02100Z9', '02100ZC', '02100ZF', '02110Z9', '211083', '211088', '211089', '021108C', '021108F', '021108W', '211093', '211098', '211099', '021109C', '021109F', '021109W', '02110A3', '02110A8', '02110A9', '02110AC', '02110AF', '02110AW', '02110J3', '02110J8', '02110J9', '02110JC', '02110JF', '02110JW', '02110K3', '02110K8', '02110K9', '02110KC', '02110KF', '02110KW', '02110Z3', '02110Z8', '02110ZC', '02110ZF', '212083', '212088',**  **'212089', '021208C', '021208F', '021208W', '212093', '212098', '212099', '021209C', '021209F', '021209W', '02120A3', '02120A8', '02120A9', '02120AC', '02120AF', '02120AW', '02120J3', '02120J8', '02120J9', '02120JC', '02120JF', '02120JW', '02120K3', '02120K8', '02120K9', '02120KC', '02120KF', '02120KW', '02120Z3', '02120Z8', '02120Z9', '02120ZC', '02120ZF', '213083', '213088', '213089', '021308C', '021308F', '021308W', '213093', '213098', '213099', '021309C', '021309F', '021309W', '02130A3', '02130A8', '02130A9', '02130AC', '02130AF', '02130AW', '02130J3', '02130J8', '02130J9', '02130JC', '02130JF', '02130JW', '02130K3', '02130K8', '02130K9', '02130KC', '02130KF', '02130KW', '02130Z3', '02130Z8', '02130Z9', , '02130ZF', '021K0Z5', '021L0Z5', '02540ZZ', '270046', '027004Z', '270056', '027005Z', '270066', '027006Z', '270076', '027007Z', '02700D6', '02700DZ', '2.70E+09', '02700EZ', '02700F6', '02700FZ', '02700G6', '02700GZ', '02700T6', '02700TZ', '02700Z6', '02700ZZ', '271046', ‘027104Z','271056', '027105Z', '271066', '027106Z', '271076', '027107Z', '02710D6', '02710DZ', '2.71E+09', '02710EZ', '02710F6', '02710FZ', '02710G6', '02710GZ', '02710T6', '02710TZ', '02710Z6', '02710ZZ', ‘272046', '027204Z', '272056',**  **'027205Z', '272066', '027206Z', '272076', '027207Z', '02720D6', '02720DZ', '2.72E+09', '02720EZ', '02720F6', '02720FZ', '02720G6', '02720GZ', '02720T6', '02720TZ', '02720Z6', '02720ZZ', '273046', '027304Z', '273056', '027305Z', '273066', '027306Z', '027307Z', '02730D6', '02730DZ', '2.73E+09', '02730EZ', '02730F6', '02730FZ', '02730G6', '02730GZ', '02730T6', '02730TZ', '02730Z6', '02730ZZ', '02B40ZX', '02B40ZZ', '02C00Z6', '02C00ZZ', '02C10Z6', '02C10ZZ', '02C20Z6', '02C20ZZ', '02C30Z6', '02C30ZZ', '02C40ZZ', '02H400Z', '02H402Z', '02H403Z', '02H40DZ', '02H40JZ', '02H40KZ', '02H40MZ', '02H40YZ', '02N00ZZ', '02N10ZZ', '02N20ZZ', '02N30ZZ', '02N40ZZ', '02Q00ZZ', '02Q10ZZ', '02Q20ZZ', '02Q30ZZ', '02Q40ZZ', '02S10ZZ', '02S00ZZ', '3E07016', '3E07017', '3E070GC', '3E070KZ', '3E070PZ'** |
| **Aortic valve surgery** | **'3511', '3521', '3522'** | **'024F07J', '024F08J', '024F0JJ', '024F0KJ', '027F04Z', '027F0DZ',**  **'02QF0ZJ', '02RF07Z', '02RF08Z', '02RF0JZ', '02RF0KZ', '02UF07J',**  **'02UF07Z', '02UF08J', '02UF08Z', '02UF0JJ', '02UF0JZ', '02UF0KJ',**  **'02UF0KZ', 'X2RF032'** |
| **Mitral valve surgery** | **'3512', '3523', '3524'** | **'024G072', '024G082', '024G0J2', '024G0K2', '025G0ZZ', '027G04Z',**  **'027G0DZ', '027G0ZZ', '02BG0ZX', '02BG0ZZ', '02CG0ZZ', '02NG0ZZ', '02QG0ZE', '02QG0ZZ', '02RG07Z', '02RG08Z', '02RG0JZ', '02RG0KZ', '02UG07E', '02UG07Z', '02UG08E', '02UG08Z', '02UG0JE', '02UG0JZ', '02UG0KE', '02UG0KZ', '02VG0ZZ', '02WG07Z', '02WG08Z', '02WG0JZ',**  **'02WG0KZ'** |
| **Tricuspid valve surgery** | **'3514', '3527', '3528'** | **'024J072', '024J082', '024J0J2', '024J0K2', '027J04Z', '027J0DZ',**  **'02QJ0ZG', '02RJ07Z', '02RJ08Z', '02RJ0JZ', '02RJ0KZ', '02UJ07G',**  **'02UJ07Z', '02UJ08G', '02UJ08Z', '02UJ0JG', '02UJ0JZ', '02UJ0KG',**  **'02UJ0KZ'** |
| **Pulmonary valve surgery** | **'3513', '3525', '3526'** | **'027H04Z', '027H0DZ', '02LH0CZ', '02LH0DZ', '02RH07Z', '02RH08Z', '02RH0JZ', '02RH0KZ', '02UH07Z', '02UH08Z', '02UH0JZ', '02UH0KZ'** |

**PAC UTILIZATION:**

| **Variable** | **ICD-9** | **ICD-10** |
| --- | --- | --- |
| **PAC** | **8963, 8964, 8966, 8967, 8968**  **89.63 = pulmonary artery pressure monitoring**  **89.64 = pulmonary artery wedge monitoring**  **89.66 = measurement of mixed venous blood gases**  **89.67 = monitoring of cardiac output by oxygen consumption technique [Fick method]**  **89.68 = monitoring of cardiac output by other technique [thermodilution indicator])** | **4A133B3**  **4A1239Z**  **02HP00Z Insertion of Pressure Sensor Monitoring Device into Pulmonary Trunk, Open Approach**  **02HP02Z Insertion of Monitoring Device into Pulmonary Trunk, Open Approach**  **02HP03Z Insertion of Infusion Device into Pulmonary Trunk, Open Approach**  **02HP0DZ Insertion of Intraluminal Device into Pulmonary Trunk, Open Approach**  **02HP0YZ Insertion of Other Device into Pulmonary Trunk, Open Approach**  **02HP30Z Insertion of Pressure Sensor Monitoring Device into Pulmonary Trunk, Percutaneous Approach**  **02HP32Z Insertion of Monitoring Device into Pulmonary Trunk, Percutaneous Approach**  **02HP33Z Insertion of Infusion Device into Pulmonary Trunk, Percutaneous Approach**  **02HP3DZ Insertion of Intraluminal Device into Pulmonary Trunk, Percutaneous Approach**  **02HP3YZ Insertion of Other Device into Pulmonary Trunk, Percutaneous Approach**  **02HP40Z Insertion of Pressure Sensor Monitoring Device into Pulmonary Trunk, Percutaneous Endoscopic Approach**  **02HP42Z Insertion of Monitoring Device into Pulmonary Trunk, Percutaneous Endoscopic Approach**  **02HP43Z Insertion of Infusion Device into Pulmonary Trunk, Percutaneous Endoscopic Approach**  **02HP4DZ Insertion of Intraluminal Device into Pulmonary Trunk, Percutaneous Endoscopic Approach**  **02HP4YZ Insertion of Other Device into Pulmonary Trunk, Percutaneous Endoscopic Approach**  **02HQ00Z Insertion of Pressure Sensor Monitoring Device into Right Pulmonary Artery, Open Approach**  **02HQ02Z Insertion of Monitoring Device into Right Pulmonary Artery, Open Approach**  **02HQ03Z Insertion of Infusion Device into Right Pulmonary Artery, Open Approach**  **02HQ0DZ Insertion of Intraluminal Device into Right Pulmonary Artery, Open Approach**  **02HQ0YZ Insertion of Other Device into Right Pulmonary Artery, Open Approach**  **02HQ30Z Insertion of Pressure Sensor Monitoring Device into Right Pulmonary Artery, Percutaneous Approach**  **02HQ32Z Insertion of Monitoring Device into Right Pulmonary Artery, Percutaneous Approach**  **02HQ33Z Insertion of Infusion Device into Right Pulmonary Artery, Percutaneous Approach**  **02HQ3DZ Insertion of Intraluminal Device into Right Pulmonary Artery, Percutaneous Approach**  **02HQ3YZ Insertion of Other Device into Right Pulmonary Artery, Percutaneous Approach**  **02HQ40Z Insertion of Pressure Sensor Monitoring Device into Right Pulmonary Artery, Percutaneous Endoscopic Approach**  **02HQ42Z Insertion of Monitoring Device into Right Pulmonary Artery, Percutaneous Endoscopic Approach**  **02HQ43Z Insertion of Infusion Device into Right Pulmonary Artery, Percutaneous Endoscopic Approach**  **02HQ4DZ Insertion of Intraluminal Device into Right Pulmonary Artery, Percutaneous Endoscopic Approach**  **02HQ4YZ Insertion of Other Device into Right Pulmonary Artery, Percutaneous Endoscopic Approach**  **02HR00Z Insertion of Pressure Sensor Monitoring Device into Left Pulmonary Artery, Open Approach**  **02HR02Z Insertion of Monitoring Device into Left Pulmonary Artery, Open Approach**  **02HR03Z Insertion of Infusion Device into Left Pulmonary Artery, Open Approach**  **02HR0DZ Insertion of Intraluminal Device into Left Pulmonary Artery, Open Approach**  **02HR0YZ Insertion of Other Device into Left Pulmonary Artery, Open Approach**  **02HR30Z Insertion of Pressure Sensor Monitoring Device into Left Pulmonary Artery, Percutaneous Approach**  **02HR32Z Insertion of Monitoring Device into Left Pulmonary Artery, Percutaneous Approach**  **02HR33Z Insertion of Infusion Device into Left Pulmonary Artery, Percutaneous Approach**  **02HR3DZ Insertion of Intraluminal Device into Left Pulmonary Artery, Percutaneous Approach**  **02HR3YZ Insertion of Other Device into Left Pulmonary Artery, Percutaneous Approach**  **02HR40Z Insertion of Pressure Sensor Monitoring Device into Left Pulmonary Artery, Percutaneous Endoscopic Approach**  **02HR42Z Insertion of Monitoring Device into Left Pulmonary Artery, Percutaneous Endoscopic Approach**  **02HR43Z Insertion of Infusion Device into Left Pulmonary Artery, Percutaneous Endoscopic Approach**  **02HR4DZ Insertion of Intraluminal Device into Left Pulmonary Artery, Percutaneous Endoscopic Approach**  **02HR4YZ Insertion of Other Device into Left Pulmonary Artery, Percutaneous Endoscopic Approach** |

**SUBGROUPS:**

| **Description** | **ICD-9** | **ICD-10** |
| --- | --- | --- |
| **Congestive heart failure** | **'39891','40201','40211','40291','40401','40403',‘40411','40413','40491','40493', ‘428’, ‘4254’, ‘4255’, ‘4256’, ‘4257’, ‘4258’, ‘4259’** | **'I099','I110','I130','I132','I255','I420', 'I425','I426','I427','I428','I429','P290'** |
| **Pulmonary hypertension** | **'4150', '4160', '4161', '4162', '4163', '4164', '4165', '4166', '4167', '4168', '4169'** | **'I27', ‘I260’** |
| **Mitral valve surgery** | **'3512', '3523', '3524'** | **'024G072', '024G082', '024G0J2', '024G0K2', '025G0ZZ', '027G04Z',**  **'027G0DZ', '027G0ZZ', '02BG0ZX', '02BG0ZZ', '02CG0ZZ', '02NG0ZZ', '02QG0ZE', '02QG0ZZ', '02RG07Z', '02RG08Z', '02RG0JZ', '02RG0KZ', '02UG07E', '02UG07Z', '02UG08E', '02UG08Z', '02UG0JE', '02UG0JZ', '02UG0KE', '02UG0KZ', '02VG0ZZ', '02WG07Z', '02WG08Z', '02WG0JZ',**  **'02WG0KZ'** |
| **Tricuspid valve surgery** | **'3514', '3527', '3528'** | **'024J072', '024J082', '024J0J2', '024J0K2', '027J04Z', '027J0DZ',**  **'02QJ0ZG', '02RJ07Z', '02RJ08Z', '02RJ0JZ', '02RJ0KZ', '02UJ07G',**  **'02UJ07Z', '02UJ08G', '02UJ08Z', '02UJ0JG', '02UJ0JZ', '02UJ0KG',**  **'02UJ0KZ'** |
| **Multiple types of cardiac surgery** | **See cardiovascular surgery table above** | **See cardiovascular surgery table above** |

**COMORBIDITIES:**

|  | **ICD-9** | **ICD-10** |
| --- | --- | --- |
| **Congestive heart failure** | **'39891', '40201', '40211', '40291', '40401', '40403', ‘40411', '40413', '40491', '40493', ‘428’, ‘4254’, ‘4255’, ‘4256’, ‘4257’, ‘4258’, ‘4259’** | **'I099', 'I110', 'I130', 'I132', 'I255', 'I420', 'I425', 'I426', 'I427', 'I428', 'I429', 'P290'** |
| **Myocardial infarction** | **'410', '412'** | **'I21', 'I22'** |
| **Aortic valve disease** | **'4241', '3950', '3951', '3952', '3953', '3954', '3955', '3956', '3957', '3958', '3959', '3960', '3961', '3962', '3963', '3964', '3965', '3966', '3967', '3968', '3969'** | **‘I35’, ‘I06’,'I080', 'Q234', 'Q235', 'Q236', 'Q237', 'Q238', 'Q239'** |
| **Mitral valve disease** | **'4240', '3949', '3940', '3941', '3942'** | **'I05', 'I08', 'I34', ‘Q232’, ‘Q233’** |
| **Tricuspid valve disease** | **'4242', '3970'** | **‘I36’, ‘I07’, 'Q224', 'Q228', 'Q229', 'I082', 'I083', 'I081'** |
| **Pulmonary valve disease** | **'4243', '3971'** | **‘I37’, ‘Q221', 'Q222', 'Q223', 'I098'** |
| **Respiratory failure** | **‘5185’ ‘51881’** | **‘J80’, ‘J960’, ‘J969’** |
| **Chronic obstructive pulmonary disease** | **'4168','4169','5064','5081','5088', ‘490’, ‘491’, ‘492’, ‘493’, ‘494’, ‘495’, ‘496’, ‘497’, ‘498’, ‘499’, ‘500’, ‘501’, ‘502’, ‘503’, ‘504’, ‘505’** | **'I278','I279','J684','J701','J703', ‘J40’, ‘J41’, ‘J42’, ‘J43’, ‘J44’, ‘J45’, ‘J46’, ‘J47’, ‘J60’, ‘J61’, ‘J62’, ‘J63’, ‘J64’, ‘J65’, ‘J66’, ‘J67’,** |
| **Pulmonary hypertension** | **'4150', '4160', '4161', '4162', '4163', '4164', '4165', '4166', '4167', '4168', '4169'** | **'I27', ‘I260’** |
| **Pulmonary vascular disease** | **'4151', '4170', '4178', '4179'** | **'I260', 'I269', 'I280', 'I281', 'I288'** |
| **Hypertension** | **'401', '402','403','404','405'** | **'I10', 'I11', 'I12', 'I13', 'I15'** |
| **Pneumonia** | **‘480’,’481’,’482’,’483’,’484’,’485’,’486’** | **‘J12’,’J13’,’J14’,’J15’,’J16’,’J17’,’J18’, ‘J82’,’J69.0’,’J69’,’J95.851’** |
| **Atherosclerotic disease** | **‘440’** | **‘I70’,’I25.1’,’I25.1’,’I67.2’,’I25.1’,’I67.2’,’I27.0’** |
| **Stroke** | **'36234', ‘430’, ‘431’, ‘432’, ‘433’, ‘434’, ‘435’, ‘436’, ‘437’, ‘438’** | **‘G45','G46', ‘I60’, ‘I61’, ‘I62’, ‘I63’, ‘I64’, ‘I65’, ‘I66’, ‘I67’, ‘I68’, ‘I69’, ‘H340’** |
| **Diabetes** | **'2504','2505','2506','2507','2500','2501','2502','2503','2508','2509'** | **'E102','E103','E104','E105','E107','E112','E113',**  **'E114','E115','E117','E122','E123','E124','E125',**  **'E127','E132','E133','E134','E135','E137','E142',**  **'E143','E144','E145','E147'**  **'E100','E10l','E106','E108','E109','E110','E111',**  **'E116','E118','E119','E120','E121','E126','E128',**  **'E129','E130','E131','E136','E138','E139','E140',** |
| **Cancer** | **‘1400’ – ‘1729’, ;1740’ – ‘1958’, ‘2000’-‘2080’, ‘2386’, ‘196’, ‘197’, ‘198’, ‘199’** | **‘C00’ – ‘D49’** |
| **Chronic liver disease** | **'07022','07023','07032','07033','07044','07054','0706','0709','5733','5734','5738','5739','V427', ‘570’, ‘571’**  **'4560','4561','4562', ‘5722’, ‘5723’, ‘5724’, ‘5725’, ‘5726’, ‘5727’, ‘5728’** | **'B18','K73','K74', 'K700','K701','K702','K703','K709','K713',**  **'K714','K715','K717','K760','K762',**  **'K763','K764','K768','K769','Z944',**  **‘I850','I859','I864','I982','K704','K711',**  **'K721','K729','K765','K766','K767'** |

**Note:** ICD-9 diagnostic and procedure codes were used for defining variables obtained from the 1999-2014 and the 2015 (Q1-Q3) National Inpatient Sample databases. ICD-10 diagnostic and procedure codes were used for defining variable obtained from the 2015 (Q4) and the 2016-2019 National Inpatient Sample databases.

**Supplemental Digital Content 2 – Tables and Figures**

**Table S.1.** Risk-adjusted Linear and Logistic Regression Models for Pulmonary Artery Catheter Receipt as a Predictor of In-Hospital Death and Hospital Length of Stay Whereby Charlson’s Comorbidity Index is replaced with specific comorbidities – 1999-2019 Nationwide Inpatient Sample (n=969,034) *

|  | **PAC use** | | |
| --- | --- | --- | --- |
|  | **In-Hospital Death**  ***Deceased vs. Alive*** | **Hospital**  **Length of Stay**  ***(days)*** | **Hospital**  **Length of Stay**  ***≥ 7 days vs. < 7 days*** |
|  | **OR (95% CI)** | **β (95% CI)** | **OR (95% CI)** |
| **RISK-ADJUSTED MODELS:** | 0.98 (0.92, 1.05) | -0.55 (-0.76, -0.34) | 0.74 (0.71, 0.79) |

***Abbreviations:*** CI = Confidence Interval; OR = Odds Ratio; PAC = Pulmonary Artery Catheter. * Adjusted for age, sex, race/ethnicity, comorbidities [congestive heart failure, myocardial infarction, aortic valve disease, mitral valve disease, tricuspid valve disease, pulmonary valve disease, respiratory failure, chronic obstructive pulmonary disease, pulmonary hypertension, hypertension, pneumonia, atherosclerotic disease, stroke, diabetes, cancer, and chronic liver disease], elective admissions, admission quarter, weekend admission status, primary payer, hospital region, hospital control, hospital location and teaching status and hospital bed size.

**Table S.2.** Unadjusted and Risk-Adjusted Linear and Logistic Regression Models for Pulmonary Artery Catheter Receipt as a Predictor of In-Hospital Death and Hospital Length of Stay After Stratifying by quartiles of hospital-level pulmonary artery catheter use – 1999-2019 Nationwide Inpatient Sample (n=969,034)

|  | **PAC use** | | |
| --- | --- | --- | --- |
|  | **In-Hospital Death**  ***Deceased vs. Alive*** | **Hospital**  **Length of Stay**  ***(days)*** | **Hospital**  **Length of Stay**  ***≥ 7 days vs. < 7 days*** |
|  | **OR (95% CI)** | **β (95% CI)** | **OR (95% CI)** |
| **UNADJUSTED MODELS:** |  |  |  |
| **Quartile 1 – < 1%** | 3.11 (2.61, 3.68) | 3.88 (3.05, 4.72) | 1.38 (1.20, 1.58) |
| **Quartile 2 – 1.5% - < 3.5%** | 2.42 (2.14, 2.73) | 2.25 (1.74, 2.77) | 1.11 (1.02, 1.22) |
| **Quartile 3 – ≥ 3.5% - < 9.2%** | 1.67 (1.51, 1.84) | 1.70 (1.25, 2.16) | 1.03 (0.96, 1.11) |
| **Quartile 4 – ≥ 9.2%** | 0.71 (0.64, 0.78) | -1.47 (-1.73, -1.20) | 0.69 (0.64, 0.74) |
| **ADJUSTED MODELS: *** |  |  |  |
| **Quartile 1 – < 1%** | 2.95 (2.48, 3.51) | 3.62 (2.83, 4.42) | 1.30 (1.15, 1.49) |
| **Quartile 2 – 1.5% - < 3.5%** | 2.35 (2.09, 2.68) | 2.09 (1.64, 2.55) | 1.09 (0.99, 1.19) |
| **Quartile 3 – ≥ 3.5% - < 9.2%** | 1.63 (1.48, 1.80) | 1.64 (1.22, 2.05) | 1.01 (0.96, 1.09) |
| **Quartile 4 – ≥ 9.2%** | 0.74 (0.68, 0.82) | -1.12 (-1.36, -0.88) | 0.74 (0.69, 0.78) |

***Abbreviations:*** CI = Confidence Interval; OR = Odds Ratio; PAC = Pulmonary Artery Catheter. * Adjusted for age, sex, race/ethnicity, Charlson’s comorbidity index, elective admissions, admission quarter, weekend admission status, primary payer, hospital region, hospital control, hospital location and teaching status and hospital bed size.

**Table S.3.** Causal Models using Targeted Maximum Likelihood Estimation for Pulmonary Artery Catheter Receipt as a Predictor of In-Hospital Death and Hospital Length of Stay by Subgroup Status – 1999-2019 National Inpatient Sample (n=969,034) *

|  |  | |
| --- | --- | --- |
|  | **In-Hospital Death** | **Hospital**  **Length of Stay**  ***≥ 7 days vs. < 7 days*** |
|  | **OR (95% CI)** | **OR (95% CI)** |
| **Average Treatment Effect (95% CI):** |  |  |
| **Quartile 1 – < 1%** | 0.0665  (0.0621, 0.0709) | 0.0488  (0.0433, 0.0546) |
| **Quartile 2 – 1.5% - < 3.5%** | 0.0487  (0.0420, 0.0553) | 0.0171  (0.0075, 0.0266) |
| **Quartile 3 – ≥ 3.5% - < 9.2%** | 0.0239  (0.0198, 0.0279) | 0.0054  (-0.0017, 0.0125) |
| **Quartile 4 – ≥ 9.2%** | -0.0098  (-0.0115, -0.0081) | -0.0513  (-0.0551, -0.0475) |
| **Marginal Odds Ratio (95% CI):** |  |  |
| **Quartile 1 – < 1%** | 2.90 (2.75, 3.05) | 1.30 (1.26, 1.34) |
| **Quartile 2 – 1.5% - < 3.5%** | 2.30 (2.11, 2.49) | 1.09 (1.04, 1.15) |
| **Quartile 3 – ≥ 3.5% - < 9.2%** | 1.63 (1.52, 1.75) | 1.03 (0.99, 1.07) |
| **Quartile 4 – ≥ 9.2%** | 0.76 (0.72, 0.80) | 0.78 (0.77, 0.80) |
| **Causal Risk Ratio (95% CI):** |  |  |
| **Quartile 1 – < 1%** | 2.70 (2.58, 2.83) | 1.07 (1.06, 1.08) |
| **Quartile 2 – 1.5% - < 3.5%** | 2.18 (2.11, 2.36) | 1.02 (1.01, 1.04) |
| **Quartile 3 – ≥ 3.5% - < 9.2%** | 1.59 (1.49, 1.70) | 1.01 (0.99, 1.07) |
| **Quartile 4 – ≥ 9.2%** | 0.77 (0.73, 0.81) | 0.93 (0.92, 0.93) |

***Abbreviations:*** CI = Confidence Interval; PAC = Pulmonary Artery Catheter. * Adjusted for age, sex, race/ethnicity, Charlson’s comorbidity index, elective admissions, admission quarter, weekend admission status, primary payer, hospital region, hospital control, hospital location and teaching status and hospital bed size.

**Table S.4.** Unadjusted and Risk-Adjusted Linear and Logistic Regression Models for Pulmonary Artery Catheter Receipt as a Predictor of In-Hospital Death and Hospital Length of Stay Among Patients with Mitral Valve Repair and Tricuspid Valve Repair and no Combined Surgeries – 1999-2019 Nationwide Inpatient Sample (n=969,034)

|  | **PAC use** | | |
| --- | --- | --- | --- |
|  | **In-Hospital Death**  ***Deceased vs. Alive*** | **Hospital**  **Length of Stay**  ***(days)*** | **Hospital**  **Length of Stay**  ***≥ 7 days vs. < 7 days*** |
|  | **OR (95% CI)** | **β (95% CI)** | **OR (95% CI)** |
| **UNADJUSTED MODELS:** |  |  |  |
| **Mitral Valve Repair (N=50,859)** | 1.04 (0.90, 1.19) | -0.82 (-1.29, -0.35) | 0.82 (0.75, 0.90) |
| **Tricuspid Valve Repair (N=5,735)** | 0.94 (0.64, 1.38) | 4.35 (1.85, 6.84) | 1.74 (1.25, 2.42) |
| **ADJUSTED MODELS: *** |  |  |  |
| **Mitral Valve Repair (N=50,859)** | 1.02 (0.89, 1.17) | -0.67 (-1.05, -0.28) | 0.77 (0.70, 0.85) |
| **Tricuspid Valve Repair (N=5,735)** | 0.71 (0.49, 1.05) | 2.39 (0.082, 4.71) | 1.06 (0.77, 1.47) |

***Abbreviations:*** CI = Confidence Interval; OR = Odds Ratio; PAC = Pulmonary Artery Catheter. * Adjusted for age, sex, race/ethnicity, Charlson’s comorbidity index, elective admissions, admission quarter, weekend admission status, primary payer, hospital region, hospital control, hospital location and teaching status and hospital bed size.

**Figure S.1.** Histogram for pulmonary artery catheter rate by hospital – 1999-2019 National Inpatient Sample (n=3,395)


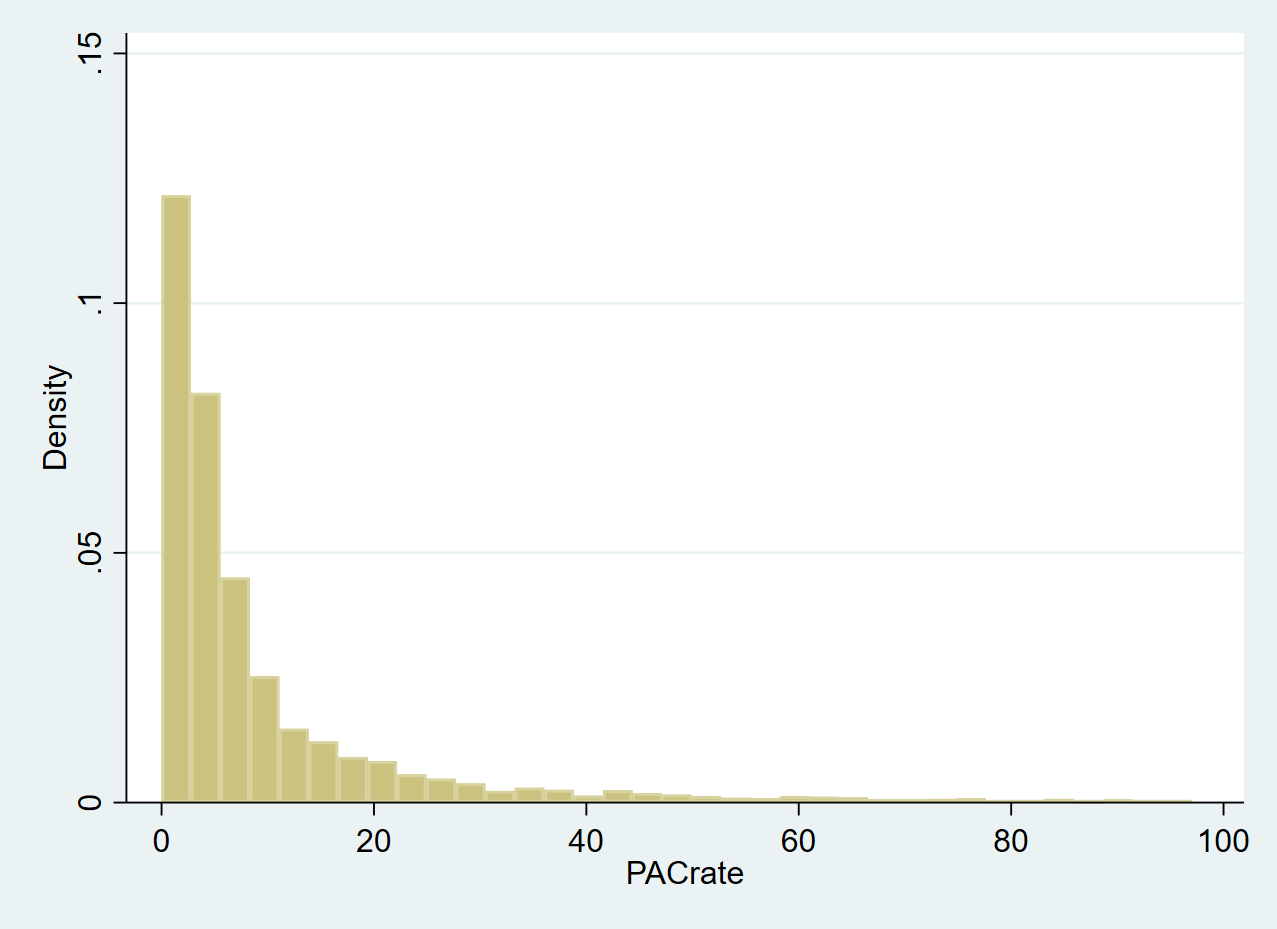


**Figure S.2.** Pulmonary artery catheter rate among cardiac surgeries by year – 1999-2019 National Inpatient Sample (n=969,034)

* Chi-square test for general association between PAC use and year of admission, revealed no statistically significant differences in PAC use among the years (P = 0.27).
